# Exploring the Diversity in Oil Content, Fatty Acid Profiles, and Seed Coat Color in Sudanese Sesame Germplasm: Implications for Breeding and Crop Improvement

**DOI:** 10.1002/pei3.70051

**Published:** 2025-04-04

**Authors:** Mohammed Elsafy, Wafa Badawi, Ali Zakaria, Tilal Sayed Abdelhalim, Mahbubjon Rahmatov, Eva Johansson

**Affiliations:** ^1^ Department of Plant Breeding Swedish University of Agricultural Sciences (SLU) Uppsala Sweden; ^2^ Matuq Research Station, Agricultural Research Corporation Matuq Sudan; ^3^ Agricultural Plant Genetic Resources Conservation and Research Center Agricultural Research Corporation Wad Medani Sudan; ^4^ Biotechnology and Biosafety Research Center Agricultural Research Corporation Shambat Khartoum North Sudan

## Abstract

Sesame, a key oilseed crop, thrives in arid environments and offers high‐quality oils. Sudan, a major producer and center of sesame genetic diversity, remains underutilized in breeding efforts. This study analyzed 87 Sudanese sesame accessions, revealing significant variations in oil content, fatty acid composition, and seed coat color. The findings highlight the potential of Sudanese germplasm for improving oil quality and broadening trait diversity in breeding programs. Oil content ranged from 32.8% to 50.2%, with oleic acid (41.3%–47.6%) and linoleic acid (35.0%–41.4%) as the predominant fatty acids, consistent with other regions. Some samples showed exceptionally high oleic acid levels. Seed coat color varied significantly, particularly in lightness (L*), but it showed no correlation with oil content or fatty acid composition. Its potential link to bioactive compounds warrants further study. Principal coordinates analysis showed no link between oil levels, fatty acid profiles, and the original collection sites. The findings highlight the breeding potential of Sudanese sesame germplasm, particularly for developing varieties with high unsaturated fatty acids, such as oleic acid, and diverse seed coat colors. Further studies across environments and genetic investigations are needed to ensure trait stability and optimize their use.

## Graphical abstract

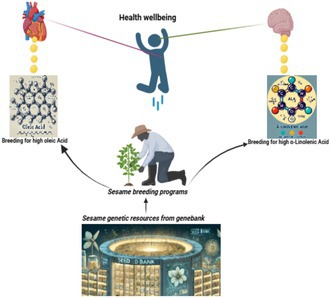

## 1 Introduction

Sesame ( *Sesamum indicum* L.) is an ancient oilseed crop of considerable economic and nutritional significance. Sesame belongs to the genus *Sesamum* in the Pedaliaceae family, which comprises approximately 38 species that thrive in tropical and subtropical regions (Kapoor et al. 2015). Although it is primarily cultivated in regions north of the equator, sesame is adaptable across latitudes between 40° N and 40° S (Dossa et al. 2016).

Archaeological evidence suggests that sesame's domestication dates back to approximately 5000 B.C. in India, with findings from the Harappa civilization supporting this timeline (Kalaiyarasi et al. 2019). By 2000 B.C., sesame had spread to Mesopotamia and the Mediterranean, becoming a crucial crop during the Bronze Age (Zech‐Matterne et al. 2015). However, identifying the exact origin of sesame is often challenging because of the complex relationship between the centers of origin and diversity. While the Indian subcontinent is acknowledged for its early domestication, East Africa, particularly Sudan, and Ethiopia, is recognized as a critical region of genetic diversity for sesame, which is crucial for improving and adapting the crop to various environments (Dossa et al. 2016; Negash et al. 2020).

Sudan, one of the leading sesame producers globally, relies on sesame as a significant agricultural and economic resource. It supports local economies, particularly in rural areas, and contributes to national export revenue (Karim and Ismai 2020). The adaptability of sesame to semi‐arid conditions enhances its food security and agricultural sustainability in regions where other crops may fail (Sabiel et al. 2015). Sesame is a valuable commodity in domestic and international markets because it has high oil content and beneficial nutritional properties. Recent efforts have been focused on improving production techniques and genetic traits to enhance yield, oil content, and overall crop resilience in Sudan (Mahmoud and Khalil 2019).

Sesame oil is known for its high content of unsaturated fatty acids, particularly oleic and linoleic acids, which contribute significant health benefits, including cardiovascular protection and anti‐inflammatory properties (Bhunia et al. 2016; Pathak et al. 2017). Some studies have indicated that the seed coat color might be correlated to the fatty acid content and composition in sesame seeds (Wang et al. 2020). The genetic variation underlying sesame seed coat color is primarily controlled by two major genes and several quantitative trait loci (QTLs), which modulate the biochemical pathways responsible for pigmentation. Studies have demonstrated that the inheritance of seed coat color is multifactorial, involving both additive and epistatic interactions between multiple genes (Du et al. 2019; Wang et al. 2020). Some genes are critical in the flavonoid biosynthesis pathway, which plays a critical role in determining pigmentation. Genetic variations within these pathways give rise to distinct seed coat phenotypes, such as black and white seeds (Wang et al. 2020). Black sesame seeds are particularly rich in antioxidants and valued for medicinal use, whereas white seeds are preferred for culinary applications (Cui et al. 2021). Breeding programs aim to increase the marketability of the crop and its nutritional value by optimizing the seed coat color (Li et al. 2021).

Despite the economic importance of sesame, its genetic diversity is threatened by conflicts and environmental challenges, particularly in Africa. The prolonged civil war in Sudan led to the loss of vital agricultural land and unique sesame germplasm (Bedigian and Harlan 1983). Several initiatives, including the establishment of gene banks and collaboration through networks such as the Eastern African Plant Genetic Resources Network (EAPGRN), have been crucial for preserving the genetic diversity of sesame (Abebe 2006). However, ongoing conflicts threaten these efforts, underscoring the urgent need for conservation, genetic resource management, and characterization of stored genotypes.

While studies have extensively examined sesame in other regions, evaluations of the Sudanese sesame germplasm (center of diversity) remain limited. This study aimed to analyze a diverse collection of Sudanese sesame gene bank accessions for oil content, fatty acid composition, and seed coat color to characterize stored accessions, evaluate present diversity within the crop in Sudan, and identify genotypes suitable for future breeding programs. Additionally, this study aimed to contribute knowledge of the importance of enhancing nutritional and market value while contributing to global conservation, documentation, and crop improvement efforts. To our knowledge, this is the first report on the fatty acid composition of sesame from Sudan and the diversity present for this trait within Sudanese germplasm.

## 2 Material and Methods

### 2.1 Plant Materials

A total of 87 Sudanese sesame accessions, preserved through long‐term conservation at the Genebank of the Agricultural Plant Genetic Resources Conservation and Research Centre (APGRC) in Sudan, were evaluated in this study. The geographical distribution and associated passport data for these accessions are detailed in Table S1 and are available on Genesys (https://www.genesys‐pgr.org/a/overview/v2AB7lOJABP).

Three widely grown Sudanese sesame cultivars, namely, Kenana‐2, Promo, and a farmer‐preferred landrace, Herheri, were included as controls for comparison, allowing for the estimation of variations in oil composition and seed coat color (Table S2). Kenana‐2, released in 1990, is an early‐maturing, drought‐tolerant sesame cultivar characterized by its large seed size and white color (Ahmed 1997). Promo, a high‐yielding, medium‐maturing cultivar released in 1998, is known for its high branching and delayed shattering ability (Ahmed 2008; Ahmed et al. 2003). From farmers' knowledge, Herheri is a high‐yielding, early‐maturing landrace characterized by its dark brown seed coat, high oil content, and drought tolerance.

### 2.2 Plant Materials and Experimental Setup

**FIGURE 1:**
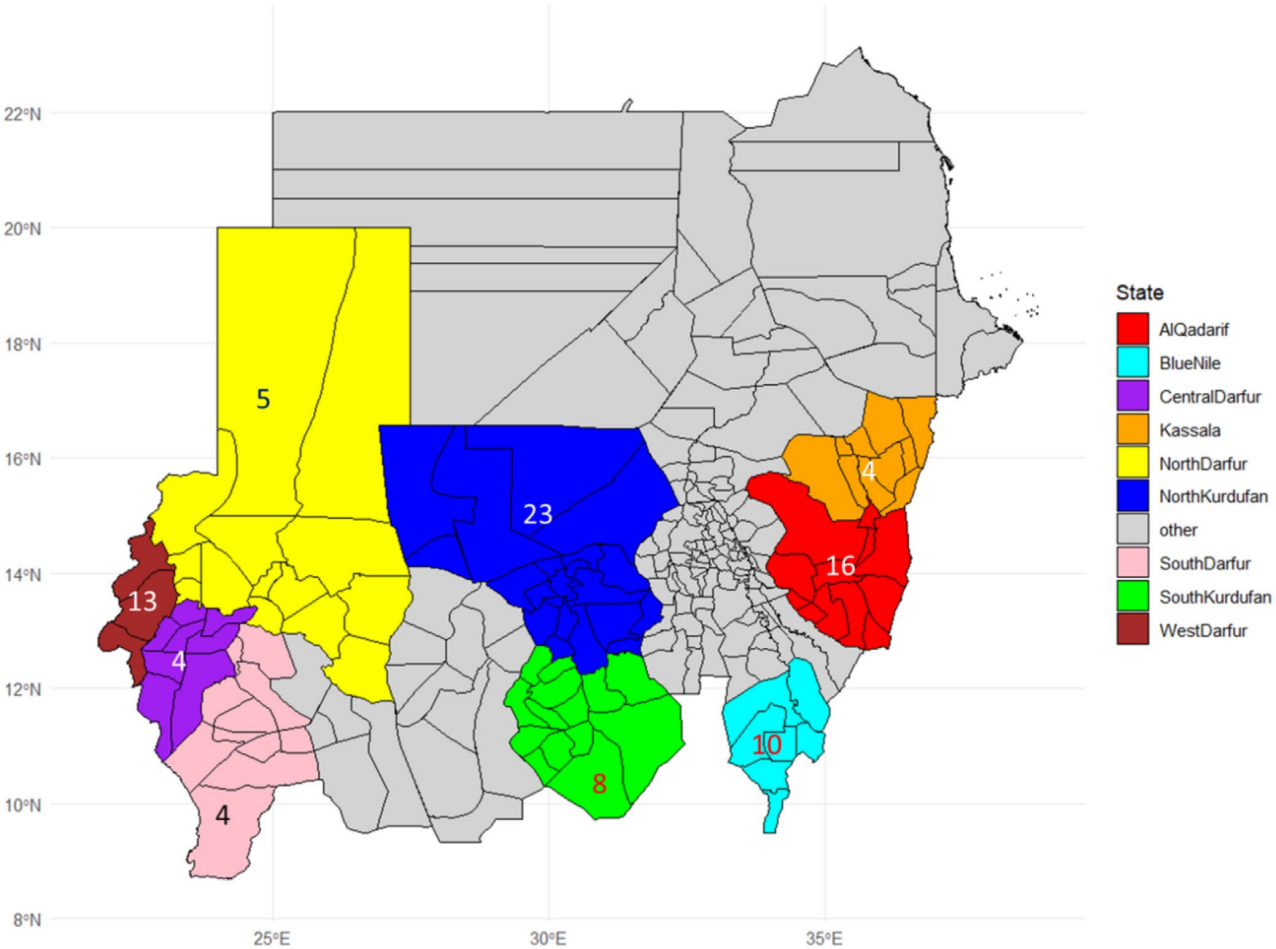
Sudan map with the number of accessions collected from different states.

This study used 87 sesame genbank accessions collected from 9 regions across Sudan, representing a wide geographical range. The highest number of accessions came from North Kordofan, with 23 accessions, followed by Gedarif (16), West Darfur (13), Blue Nile (10), South Kordofan (8), North Darfur (5), Kassala (4), Central Darfur (4), and South Darfur (4) (Figure 1 and Table S3). According to the characterization data from the Genbank of these accessions, the seed coat color of accessions varied from white, light brown, reddish brown, brown, and dark brown to gray and black. In addition, the 1000 seed weight varied from 2 to 6 g, and the days to 50% flowering ranged from 32 to 76 days (Table S1).

The selected accessions for this study were grown in the field during the 2022–2023 season at the Matouq Research Station (latitude 14°11′10″ N, longitude 32°34′48″ E) of the Agricultural Research Corporation (ARC), Sudan. The soil at the site is a vertisol, characterized by deep cracking and high clay content.

The 87 sesame accessions were sown using an Augmented Block Design (ABD) across five blocks, incorporating three standard varieties, Kenana‐2, Bromo, and Herheri, as national checks. Each block consisted of 17 entries and three checks, which were randomized within each block. Each accession was sown in a 5‐m row, spaced 0.7 m apart. Seeds were drilled at a rate of 3 kg/ha. Three weeks after sowing, the crop was thinned to one plant per hole with an inter‐row spacing of 7.5 cm.

At maturity, three random plants from each experimental plot were selected for colorimetric measurement, oil content analysis, and fatty acid composition profiling. All accessions were carefully harvested, cleared of debris, winnowed, and stored at room temperature until laboratory analysis.

### 2.3 Extraction of Lipids and Gas Chromatography

Total lipids were extracted and analyzed following the method described by Tesfaye et al. (2024), with minor modifications. Lipids were extracted from 10 seeds per genotype, with two technical replicates for each sample. The seeds were homogenized in 1 mL of 0.15 M acetic acid and 3.75 mL of a 2:1 (v/v) methanol/chloroform mixture using an IKA T18 basic ULTRA TURRAX homogenizer in a glass test tube. Subsequently, 1.25 mL of chloroform and 0.9 mL of Millipore‐grade H_2_O were added, and the samples were vortexed for 10 s. The mixture was then centrifuged at 3000 rpm for 2 min.

After centrifugation, 200 μL of the lower chloroform phase was transferred into a clean glass screw‐capped tube. The chloroform was evaporated by placing the tubes on a heated sand bed (70°C) under a stream of nitrogen gas. Once evaporation was complete, the samples were reconstituted in 100 μL heptane and methylated by adding 2 mL of methylation solution (2% concentrated H_2_SO_4_ in anhydrous methanol). The methylation reaction was performed in a sealed tube at 90°C for 1 h. After the reaction, the samples were cooled to room temperature, and 1 mL of Millipore‐grade H_2_O and 0.75 mL of heptane were added. The samples were vortexed for 15 s and centrifuged at 3000 rpm for 2 min. After centrifugation, 100 μL of the upper heptane phase, containing the fatty acid methyl esters (FAMEs), was transferred to a gas chromatography (GC) vial with a glass insert for GC analysis.

Fatty acid profiles were generated for each sample using an Agilent Model 886 gas chromatography instrument, and data were collected using a flame ionization detector (FID). Separation of the FAMEs was achieved using a WCOT fused‐silica CP‐wax 58 capillary column (50 m × 0.32 mm, Agilent) with a split ratio of 10:1. The oven temperature was set to 150°C for 0.2 min, then increased by 4°C/min to 210°C, followed by an increment of 10°C/min to 250°C. The temperature was maintained at 250°C for 5 min to ensure full elution of the samples.

The identity of each fatty acid was determined by comparing the retention times of its respective FAMEs to those a certified Me63 external standard (Larodan, Sweden). The oil content, total triacylglycerols (total TAG), total glyceride (total Gly), and percentages of fatty acidwere calculated using the following equations:

$$\text{Oil Content}\;\left(\%\right)=\left(\frac{\text{Total Weight of Fatty Acids}}{\text{Initial Weight of Seeds}}\right)\times 100$$

$$\text{Total}\;\mathrm{TAG}\;\left(\mathrm{mg}\right)=\sum \text{Concentration of individual TAGs}\;\left(\mathrm{mg}\right)$$

$$\begin{array}{c} \text{Total Glyceride}\;\left(\mathrm{mg}\right)\\ =\left(\mathrm{Sum}\;\text{of}\;\mathrm{TAG},\mathrm{DAG},\mathrm{MAG}\;\left(\mathrm{mg}\right)\times \frac{\text{Molecular Weight of Glycerol}}{\text{Molecular Weight of}\;\mathrm{TAG}\;\text{and}\;\mathrm{DAG}}\right) \end{array}$$

$$\text{Percentage of Fatty Acid}\;\left(\mathrm{FA}\right)=\left(\frac{\text{Peak Area of}\;\mathrm{FA}}{\sum \text{Peak Areas of}\;\mathrm{all}\;\mathrm{FAs}}\right)x100$$

This calculation of oil content ensures that the total fatty acids measured are expressed as a percentage of the initial seed weight, providing an accurate and precise measure of the oil content in the seeds.

### 2.4 Seed Color Measurement

A colorimeter (Chroma Meter CR 400, Minolta, Japan) was used to measure the color of the stored sesame seeds, following the method described by Hassan et al. (2021). The color parameters measured were L* (lightness), a* (red‐green), and b* (yellow‐blue). The device was calibrated using a standard white reflector plate. Two technical replicates were performed for each measurement using a Petri dish filled with 50.0 g of sesame seeds. The same seeds from these replicates were used for lipid analysis.

### 2.5 Statistical Analysis

All the statistics analyses were conducted using the open‐source R environment, version 4.3.2. Pearson's correlation coefficients were calculated to examine relationships between variables (Sedgwick 2012). The correlations were computed using the “cor” function in R, and their significance was assessed using the “cor.test” function. The resulting correlation matrix and *p*‐values provided insight into the strength and direction of linear relationships between the variables (Ornella et al. 2014). Principal Coordinates Analysis (PCoA) was employed to explore the similarity or dissimilarity among samples based on a distance matrix. The “cmdscale” function in R was used to perform classical multidimensional scaling on the distance matrix obtained from the “dist” function. The PCoA plot presents the samples in a reduced dimensional space, where the axes represent the principal coordinates that capture the maximum variation in the data.

Individual linear regression analyses were performed to assess the relationship between oil content and color parameters (L*, a*, and b*). The regression models were constructed using Oil Content (%) as the predictor variable and each color parameter as the response variable. The models were fitted using the “lm” function from the *stats* package in R, and model diagnostics were conducted using the “broom” package (Robinson 2014). The regression equations are as follows:

$$L^{*}=\beta_{0}+\beta_{1}\times \text{oil content}\;\left(\%\right)+\epsilon$$

$$a^{*}=\beta_{0}+\beta_{1}\times \text{oil content}\;\left(\%\right)+\epsilon$$

$$b^{*}=\beta_{0}+\beta_{1}\times \text{oil content}\;\left(\%\right)+\epsilon$$

where *β*_0_ is the intercept, *β*_1_ is the coefficient for (L*, a*, b*), respectively, and ϵ is the error term.

## 3 Results

### 3.1 Oil Content and Fatty Acid Composition Profiling

The oil content ranged from 32.8% to 50.2%, with a mean of 41.5% (Table S3). The total TAG varied from 7.4 to 18.3 mg, with an average of 13 mg, while total Gly content varied from 0.3 to 0.8 mg, with an overall mean of 0.6 mg. The oil composition differed among the evaluated samples (Table S3). The palmitic acid (16:0) ranged from 10.8% to 8.94% for the saturated fatty acids, with an overall average of 10.0%. Stearic acid (18:0) concentrations ranged from 6.25% to 7.80%, with a mean of 6.80%, while the arachidic acid content (20:0) ranged from 0.73% to 0.49%, with a mean value of 0.60%. For unsaturated fatty acid compositions, oleic acid (18:1) varied from 41.3% to 47.6%, with an overall mean of 44.4%. For linoleic acid (18:2), the content ranged from 41.4% to 35.0%, with a mean of 37.8%. The linolenic (18:3) acid content ranged from 0.31% to 0.14%, with a mean of 0.20%. Gadoleic acid (20:1) content varied from 0.13% to 0.03%, with a mean of 0.08%.

The range of oil content (32.8%–50.2%) in the Sudanese germplasm corresponds well with results from previous studies, including Mondal (2010) (36.3%–52.7%), Uzun (2008) (43.2%–59.0%), Were (2006) (41.7%–55.5%), and Kurt (2018) (42.6%–57.8%) (Table 1). For palmitic acid (16:0), levels ranged from 8.94% to 10.8% in our study, consistent with Kurt (2018) (8.19%–10.26%) and Uzun (2008) (8.0%–10.3%) but slightly narrower than Mondal (2010) (9.1%–14.8%). Stearic acid (18:0) ranged from 6.25% to 7.80% in this study, higher than Uzun (2008) (2.1%–4.8%) and closer to Kurt (2018) (4.63%–6.35%). Oleic acid (18:1) ranged from 41.3% to 47.6% in our study, exceeding the ranges reported by Uzun (2008) (29.3%–41.4%) and Were (2006) (35.1%–42.1%) but aligned with Kurt (2018) (36.13%–43.63%) and Mondal (2010) (36.7%–52.4%). Linoleic acid (18:2) ranged from 35.0% to 41.4% in our results, and it is within the ranges reported by Uzun (2008) (40.7%–49.3%), Were (2006) (41.9%–48.0%), and Kurt (2018) (39.13%–46.38%). Linolenic acid (18:3) ranged from 0.14% to 0.30% in this study, which is in line with Kurt (2018) (0.28%–0.40%). Arachidic acid (20:0) and gadoleic acid (20:1) were detected at 0.49%–0.7% and 0.03%–0.10% in this study but were missing in the other studies (Table 1).

**TABLE 1:** A range comparison of oil content and fatty acid composition for this study and the previous studies.

Traits	This study (%)	Mondal (2010) (%)	Uzun (2008) (%)	Were (2006) (%)	Kurt (2018) (%)
Oil content	32.8–50.2	36.3–52.7	43.2–59.0	41.7–55.5	42.6–57.8
Palmitic (16:0)	8.94–10.8	9.1–14.8	8.0–10.3	8.4–10.5	8.19–10.26
Stearic (18:0)	6.25–7.8	Not specified	2.1–4.8	4.5–6.0	4.63–6.35
Oleic (18:1)	41.3–47.6	36.7–52.4	29.3–41.4	35.1–42.1	36.13–43.63
Linoleic (18:2)	35.0–41.4	30.4–51.6	40.7–49.3	41.9–48.0	39.13–46.38
Linolenic (18:3)	0.14–0.3	NS	NS	NS	0.28–0.40
Arachidic (20:0)	0.49–0.7	NS	NS	NS	NS
Gadoleic (20:1)	0.03–0.1	NS	NS	NS	NS

### 3.2 Colorimetric Measurement

The color parameter L* ranged from 34.05 ± 0.50 to 79.8 ± 0.42, with an overall mean of 59.6 (Table S2). a* values varied from 4.55 ± 0.07 to 15.1 ± 0.00, with an overall mean of 8.4. Similarly, the b* values ranged from 12.2 ± 0.14 to 24.9 ± 0.99, with an overall mean of 18.2.

**TABLE 2:** Linear regression analysis of oil content and seed coat color parameters in Sudanese sesame gene bank collection samples.

Response_Variable	Intercept	Slope	*R* ^2^	*p*_value_Intercept	*p*_value_Slope
L*	46.27	0.32	0.01	7.71E‐06	0.18
a*	11.98	−0.09	0.02	3.59E‐09	0.06
b*	18.58	−0.01	0.00	3.64E‐14	0.87

### 3.3 Correlations and Regressions Among the Fatty Acid Composition and Colorimetric Traits

**FIGURE 2:**
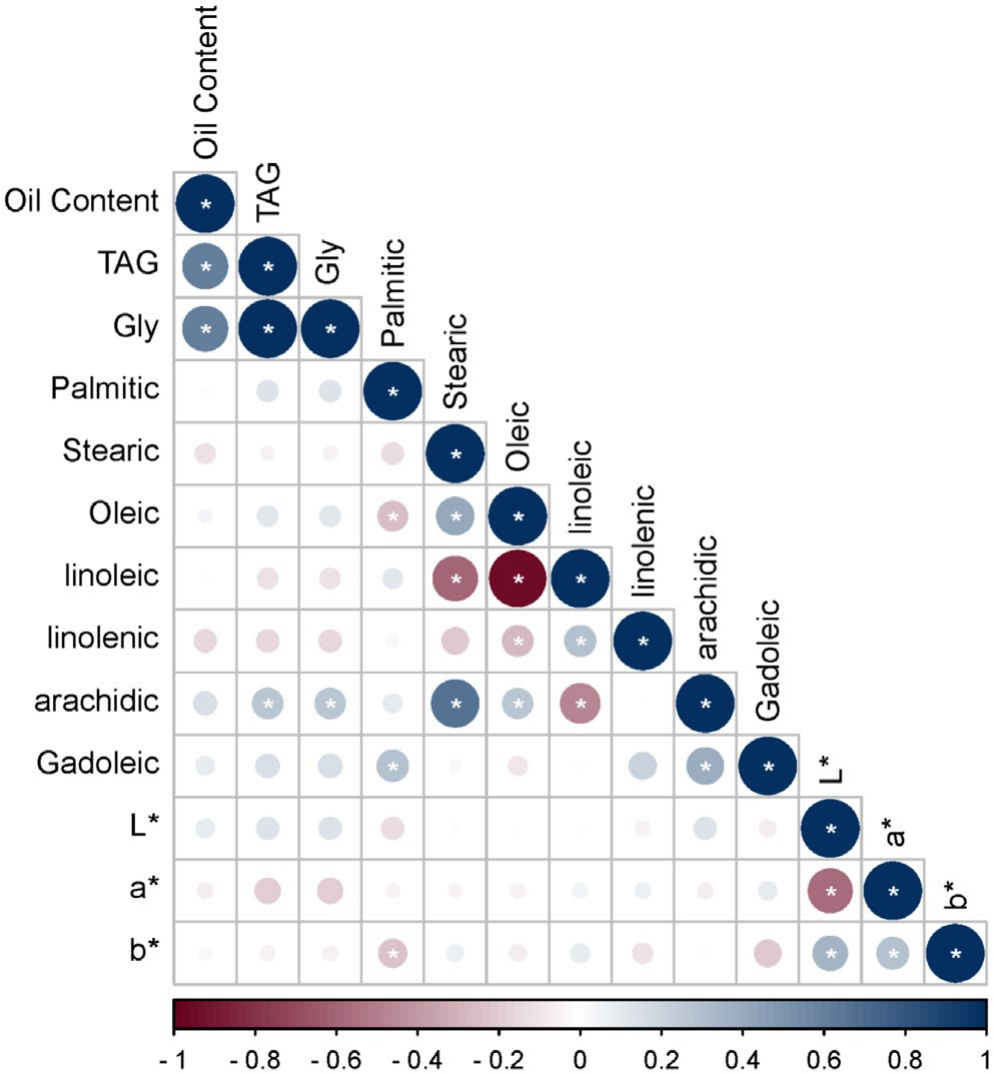
Correlation heatmap of oil content, fatty acid composition, and color parameters in sesame accessions. * indicated significance at *p* < 0.05.

The total oil content exhibited a significant positive correlation with triacylglycerols (TAG) (*r* = 0.91, *p* < 0.01). A significant negative correlation was observed between oleic acid (18:1) and linoleic acid (18:2) (r = −0.88, *p* < 0.01) (Figure 2). Palmitic acid (16:0) displayed a significant positive correlation with stearic acid (18:0) (*r* = 0.62, *p* < 0.05). Stearic acid (18:0) also showed a significant positive correlation with arachidic acid (20:0) (*r* = 0.54, *p* < 0.05). Among minor fatty acids, linolenic acid (18:3) was positively correlated with linoleic acid (18:2) (*r* = 0.46, *p* < 0.05). No significant correlations (*p* < 0.05) were found among oil and fatty acids content and composition with seed coat color parameters (Figure 2).

The regression analysis showed that the intercepts for all color parameters (L*, a*, and b*) were highly significant (*p* < 0.001), indicating their strong baseline values (Table 2). However, the slopes were largely non‐significant, with minimal variation explained (*R* ^2^ ≤ 0.02), suggesting weak or negligible relationships between the predictors and the response variables.

**FIGURE 3:**
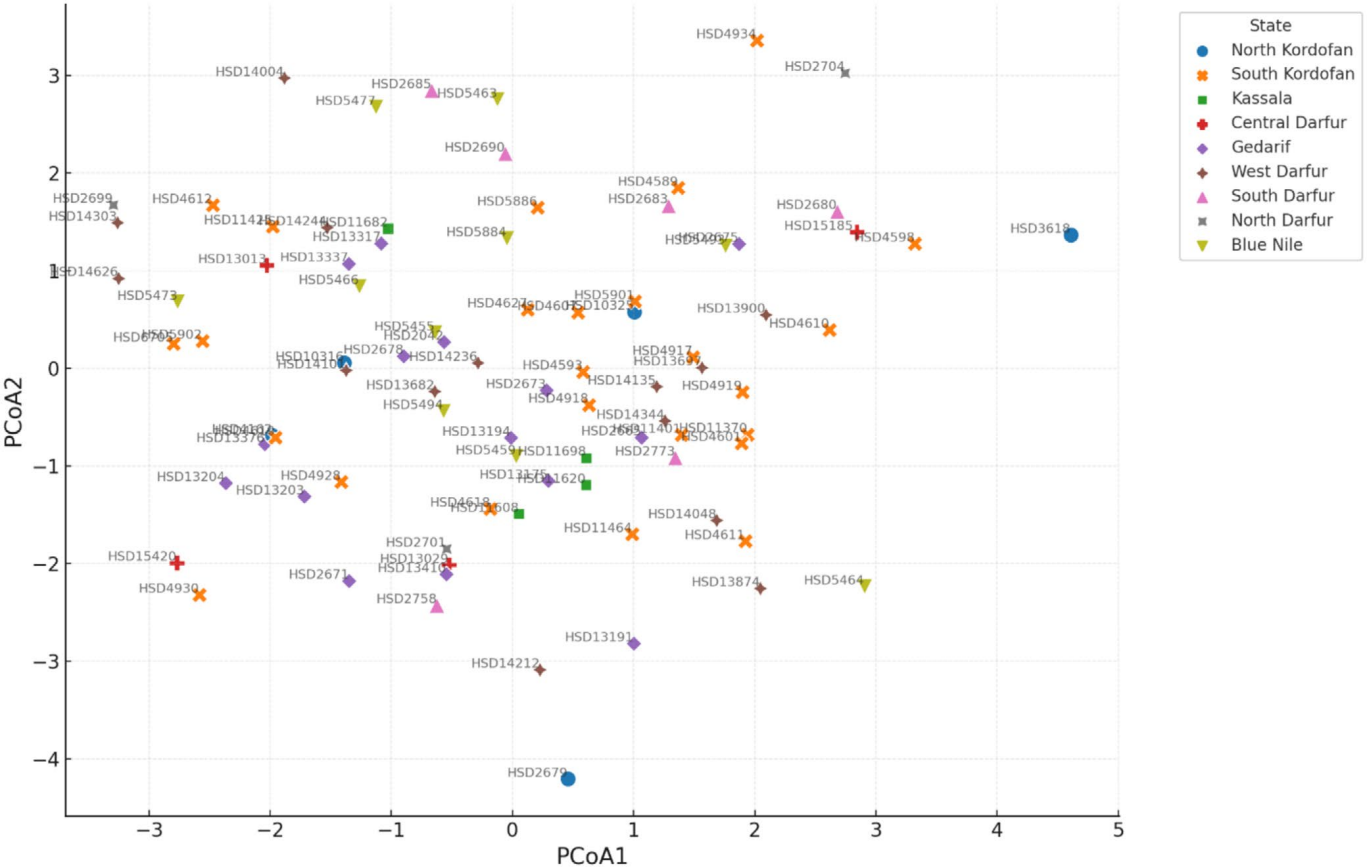
Principal coordinates analysis cluster plot based on oil content, fatty acids composition, and Sudanese sesame accessions. Collection sites of the various accessions are displayed by different colors, as explained with the symbols.

The Principal Coordinate Analysis (PCoA) revealed limited clustering patterns among sesame accessions based on their geographic origin. However, the accessions from South Kordofan formed a loose cluster, with most accessions showing positive values on PCoA1, indicating a possible similarity among the accessions from this region. The accessions from other states, for example, from North Kordofan, Kassala, and Blue Nile, were more dispersed across the plot, reflecting great variability of accessions from these regions (Figure 3). Notably, an overlap was observed between accessions from different states. However, some accessions from North Kordofan and Gedarif were positioned far from the main clusters, indicating their possible unique set of oil content, fatty acids composition, and seed coat color characteristics.

## 4 Discussion

The present study, investigating the range of oil content, fatty acids composition, and seed coat color in Sudanese sesame, revealed a highly significant variation in these traits and the presence of high levels, especially in oleic acid content. As these traits are essential for sesame breeding and crop improvement strategies, the Sudanese germplasms are of high importance to be utilized by plant breeders in Sudan and beyond.

The present study revealed that Sudanese sesame holds a significant range in total oil, total Gly, and total TAG contents. The Gly and TAG levels in sesame are important concerning human health and nutrition, as these compounds contain essential fatty acids supporting metabolic functions and overall health (Carino and Vital 2022). Previous studies have indicated that enhancing the TAG content in crops can increase beneficial fatty acids essential for preventing chronic diseases (Saini et al. 2020). Furthermore, TAG composition affects the shelf life of oils as well as their suitability for cooking (Zeb and Ahmad 2017). Additional studies are recommended to understand the performance of Gly and TAG contents in Sudanese sesame across diverse environments to assess their stability and adaptability. In addition, genomic analyses, including genome‐wide association studies (GWAS) and quantitative trait loci (QTL) mapping, can potentially elucidate the genetic basis of oil content and connect desirable agronomic traits.

The large range of content of unsaturated fatty acids, i.e., oleic, linoleic, and α‐linolenic acids in the Sudanese sesame, and specifically, the high levels of oleic acid (up to 46.7%) contribute significantly to their specificity and potential to be used in plant breeding. These fatty acids are essential for human health and biofortification; several studies have highlighted their importance. Thus, oleic acid has been shown to reduce low‐density lipoprotein (LDL) cholesterol levels and the risk of cardiovascular diseases (Kamdar et al. 2021), but it is also useful in breeding for oxidative stability (Bahkali et al. 1998). Linoleic acid is essential for maintaining skin health and regulating immune function (Kim et al. 2014). Additionally, it has been demonstrated that alanine is an omega‐3 fatty acid that lowers blood pressure and improves lipid profiles, helping to reduce the risk of heart disease (Han et al. 2019; Wu et al. 2020). Thus, enriching crops with these fatty acids contributes to improved nutritional quality and health benefits Siahbalaci et al. (2021), and for this, the incorporation of high‐content genotypes in plant breeding programs might be an important tool. Previous studies have shown that the fatty acid profile of sesame oil exhibits significant variation connected to the environmental difference in production (Zahran et al. 2020), which indicates the importance of environmental and genetic factors in determining the fatty acid composition in sesame. Specific genes, such as the FAD2, are involved in synthesizing fatty acids determining the oleic‐to‐linoleic acid ratios (Wei et al. 2015). A comprehensive understanding of the genetic basis underlying the high oleic acid content observed in some of the accessions evaluated in this study, along with insights into the stability of this trait across different environmental conditions, is crucial for leveraging these accessions in sesame breeding programs. Such knowledge would enhance efforts to develop improved sesame varieties with superior oil quality, benefiting breeding initiatives in Sudan and potentially contributing to global sesame improvement. Also, for the saturated fatty acids, a high variability was found in the present study, in particular for palmitic, stearic, and arachidic acids. Saturated fatty acids are known to impact human health negatively, contributing to increased cholesterol levels and cardiovascular disease risk (Sultan 2018). However, the saturated fatty acids also play a significant role in the oxidative stability of the oil, thereby enhancing oil shelf life, and making them critical factors in oil formulation and processing (Hassanien et al. 2014). Among the saturated fatty acids in sesame, arachidic acid is of specific interest for certain food and cosmetics products (Cerone and Smith 2021). Arachidic acid was detected in relatively low concentrations (less than 1%) in sesame seeds analyzed in this study. Previous research has indicated that arachidic acid has a less significant negative effect on human health as compared to other saturated fatty acids, such as palmitic and stearic acids (Mthana et al. 2022). Genetic factors and environmental conditions have been shown to influence the levels of arachidic acid (Abd‐Elhafeez et al. 2020) indicating a need to further understand the mechanisms of synthesis of this fatty acid.

The sesame accessions in the present study showed a large variability in seed coat color. Sesame seed coat color has in previous studies been linked to (i) taste and qualitative aspects when used in food formulations, where often light coat color is preferred (Cui et al. 2021; Pandey et al. 2013), (ii) biochemical functions that influence oil metabolism and disease resistance (Zhang et al. 2013), and (iii) nutritional aspects including the content of bioactive components, where often dark colored types hold a higher content (Dossou et al. 2021). This study did not identify any correlation between seed coat color and oil content, fatty acid content, or fatty acid composition, as has been reported in some previous studies (Uzun and Çağırgan 2006). Additionally, in Sudan, a prevalent belief among sesame farmers associates dark brown seed color with higher oil content (ETI 2023). Therefore, it is crucial to implement Farmer Field Schools (FFS) focused on sesame agronomy and seed characteristics to address such misconceptions. The variation in seed coat color observed among the Sudanese accessions presents new opportunities for breeding programs. For example, dark‐colored seeds, potentially associated with higher lignin content, could be incorporated into breeding efforts to target specific traits of interest. Previous studies have indicated that dark seed coats are associated with a high lignan content, which enhances the oil antioxidant properties of sesame (Comini et al. 2023). Lignans are known to act as a *secoisolariciresinol diglucoside*, which enhances the activity of antioxidant enzymes such as superoxide dismutase and catalase (Pilar et al. 2017; Rajesha et al. 2006), thereby reducing inflammation and oxidative stress.

Additionally, lignans may contribute to cardiovascular health by decreasing C‐reactive protein (CRP), a marker of inflammation (Bolvig et al. 2017). However, the connection between seed coat color and lignan content in the Sudanese accessions has to be further elucidated, as such studies have not been performed on Sudanese sesame. Also, in some ancient sesame germplasm, lignan content has been found to correlate with oil content, with white‐seeded varieties showing higher lignan levels compared to dark‐seeded germplasm (Kancharla and Arumugam 2020).

Consistent with previous studies (Kurt 2018), the present study found a significant negative correlation between oleic and linoleic acid. This relationship is attributed to the desaturation of oleic acid into linoleic acid, as Relina et al. (2022) reported.

The PCoA cluster plot indicated a large variation among the collected Sudanese sesame accessions, although it did not show any clear clustering pattern based on the regions of their collection However, the majority of the accessions from South Kordofan were found to have positive PCoA values, thereby forming a loose cluster. This indicates that the samples collected from this region might vary more similarly regarding oil, fatty acid, and composition than accessions from other regions. Also, the PCoA1 was found to explain a high degree of the variation, indicating that the spread of the accessions along this axis described the majority of the variation among the samples. It is well known that historical seed migrations and trade have shaped the genetic landscape of sesame, with accessions originating from Europe, Asia, and North America forming different groups when analyzed by double‐digest site‐associated DNA sequencing (Basak et al. 2019). The lack of clustering by accessions from different regions of Ethiopia indicates that sesame seeds have been traded across the country and that there are significant variations in oil content and fatty acid composition of origin other than regional. Thus, an increased understanding of the background for the variation in Sudanese sesame as regards oil content, fatty acids content, and composition, similar to what has been studied previously, sequencing a wide collection of more than 700 accessions collected globally (although only one representing the Sudanese gene pool; Wei et al. 2015), will contribute to improved opportunities in breeding high‐quality sesame varieties for Sudan and beyond.

## 5 Conclusions

Sudanese sesame accessions exhibit extensive variation in oil content, fatty acid composition, and seed coat color traits that are essential for breeding programs to improve oil quality and nutritional value in sesame. The observed diversity in fatty acid profiles, particularly the presence of high oleic acid content in certain accessions, provides valuable opportunities for breeders to enhance the nutritional attributes of sesame oil. Incorporating multiple traits into breeding efforts is critical to optimizing oil yield and quality. The significant variation observed within Sudanese sesame highlights the importance of utilizing these resources in breeding programs, not only in Sudan but also in global initiatives. Despite Sudan's status as a center of sesame diversity, the Sudanese gene pool remains underutilized in international breeding due to the country's unstable political and economic conditions in recent decades. However, with the increasing pressures of climate change and the need to feed a growing global population, every available genetic resource must be considered for breeding efforts. Preserving and utilizing the genetic diversity of Sudanese sesame is essential for developing high‐quality, resilient cultivars capable of thriving in diverse environmental conditions. These efforts will contribute to food security and the sustainability of sesame production on both local and global scales.

## Conflicts of Interest

The authors declare no conflicts of interest.

## Supporting information


Data S1.

## Data Availability

The data supporting the findings of this study are available from the corresponding author upon reasonable request.
